# Study on the Function of *ID2* Gene in Granulosa Cells of Ovaries of Hetian Sheep and Its Correlation Analysis with Lambing Traits

**DOI:** 10.3390/ani15223271

**Published:** 2025-11-12

**Authors:** Huiping Sun, Xinkun Wang, Lexiao Zhu, Gul Muhammad Shahbaz, Ruohuai Gu, Qiaoyan Huang, Wei Li, Feng Xing

**Affiliations:** 1 College of Animal Science, Tarim University, Alar 843300, China; sunhuiping223@163.com (H.S.); wdaqqbt@163.com (X.W.); 18538512047@163.com (L.Z.); shahbazbalouch42@gmail.com (G.M.S.); a18346865515@163.com (R.G.); huangqy0316@163.com (Q.H.); 18597814328@163.com (W.L.); 2 Key Laboratory of Tarim, Animal Husbandry Science and Technology, Xinjiang Production & Construction Corps, Alar 843300, China

**Keywords:** Hetian sheep, granulosa cells, *ID2* gene, single nucleotide polymorphism, reproductive efficiency, ovary

## Abstract

Reproductive efficiency is a key determinant of sheep productivity, yet Hetian sheep remain limited by relatively low fecundity despite their adaptability to harsh environments. The inhibitor of DNA binding 2 (ID2) gene is known to regulate cell proliferation and differentiation, but its specific role in sheep reproduction is not well understood. This study aimed to characterize the *ID2* gene in Hetian sheep and to assess its functional association with ovarian granulosa cells and litter size. The coding sequence of ovine *ID2* was cloned and analyzed using bioinformatics tools. Tissue-specific expression patterns were measured by quantitative PCR at different pubertal stages. A total of 157 ewes were genotyped to identify single-nucleotide polymorphisms (SNPs) and their association with litter size. Functional studies were performed by lentiviral overexpression of ID2 in granulosa cells, with effects evaluated using CCK-8 proliferation assays, ELISA for hormone secretion, and RT-qPCR for related gene expression. *ID2* was highly expressed in the ovary, particularly during puberty. Four SNPs (g.18202368 A>T, g.18202372 G>A, g.18202431 G>C, g.18202472 G>C) were significantly associated with increased litter size. Overexpression of ID2 promoted granulosa cell proliferation, increased progesterone, decreased estradiol, and altered expression of key genes in the TGF-β/BMP-SMAD signaling pathway. The *ID2* gene plays a crucial role in ovarian function and reproductive regulation in Hetian sheep. Its polymorphisms and functional impact on granulosa cells suggest that ID2 is a promising candidate gene for marker-assisted selection to improve reproductive efficiency in sheep.

## Simple Summary

Sheep are an important source of meat, wool, and income for farmers, but some breeds, such as the Hetian sheep in Xinjiang, produce only a small number of lambs each year. Improving their ability to have more lambs would benefit farmers and local communities. In this study, we focused on a gene called *ID2*, which helps control how cells grow and develop. We found that this gene is especially active in the ovaries of Hetian sheep, where it likely influences fertility. By studying the DNA of 157 sheep, we discovered several genetic differences in *ID2* that were linked to larger litter sizes. We also tested how the gene works in ovarian cells and found that it makes these cells grow faster and changes the levels of important reproductive hormones. These findings show that *ID2* plays an important role in sheep reproduction. This information can help scientists and breeders develop better strategies to select sheep with stronger fertility traits, leading to improved flock productivity and better support for the sheep industry.

## 1. Introduction

Hetian sheep are a wool breed indigenous to the Hetian region of Xinjiang and constitute one of the region’s most important livestock resources. They tolerate heat, sparse feed, and disease, and have been placed by China’s Ministry of Agriculture on the National List of Protected Livestock and Poultry Genetic Resources. Ewes typically reach puberty at approximately 8–12 months and are first bred at 18–24 months, with a reported lambing percentage of about 102.52% [1]. Because fecundity remains modest, improving litter size is a priority breeding objective with clear economic benefits for local communities.

Lambing performance integrates multiple reproductive processes—endocrine regulation, folliculogenesis, ovulation, fertilization, embryo implantation, and placental/fetal development [2,3]. Both among-breed and within-breed genetic variation for these traits is substantial and generally sufficient to support steady selection responses; consequently, optimizing genetic potential for reproduction is central to enhancing flock productivity [4].

ID2 (inhibitor of DNA binding 2) belongs to the ID protein family, characterized by a helix–loop–helix (HLH) structure lacking a basic DNA-binding domain. The ID2 protein has two typical biological characteristics: inhibiting cell differentiation and promoting cell proliferation. Granulosa cells (GCs) are pivotal for follicular development: their proliferation, differentiation, and steroidogenesis determine oocyte support and follicle fate [5]. GCs’ number and function are governed by networks of genes and signaling pathways, including inhibitor of DNA binding 2 (ID2) [6], bone morphogenetic protein receptor type 2 (*BMPR2*) [7], anti-Müllerian hormone (*AMH*) [8], the PI3K/AKT axis [9], and BMP/SMAD cascades [10]. Transforming growth factor-β (TGF-β) signaling coordinates intercellular communication to modulate transcription, cell-cycle progression, differentiation, adhesion, migration, apoptosis, and extracellular-matrix dynamics [11,12]. For example, Xing Du et al. showed that TGF-β signaling interacts with FSHR pathways and regulates ovarian GCs apoptosis via miR-143 [13]. As a downstream effector within TGF-β networks, ID2 has been implicated in GCs proliferation and follicular development. Across species, ID2 is involved in reproductive regulation; for instance, its induced expression in rat Sertoli cells [14]. ID2 participates in Sertoli-cell differentiation and endocrine regulation and is enriched in chicken ovarian GCs and testicular Sertoli cells [15,16]; it is upregulated in bovine follicles [17] and shows highest expression in fully differentiated porcine GCs and cumulus–oocyte complexes [18] and ID2-deficient female mice exhibit severe lactation defects with decreased mammary epithelial proliferation [19], collectively supporting a plausible role for ID2 in follicular dynamics and fecundity.

With the advent of whole-genome resequencing, selection-signal scans (e.g., F_ST_ and π ratio) have become powerful tools to detect genomic regions under differential selection between single- and multiple-lambing groups. Through within-population comparisons of Hetian sheep, 290 shared selected genomic regions were identified, and 332 candidate genes were annotated in both single-lambing (SLE) and twin-lambing (TLE) groups; *ID2* was among 13 genes significantly enriched in pathways related to lambing rate (including TGF-β signaling). Genetic studies of ovine fecundity frequently focus on SNPs in candidate genes [20,21]; for example, Zhang et al. reported that T/T and T/G genotypes at rs400827589 in Small-Tailed Han sheep were associated with greater litter size than G/G (*p* < 0.05) [22]. However, no published data directly link *ID2* to lambing number in Hetian sheep.

To address this gap, we cloned the coding sequence (CDS) of ovine *ID2* from Hetian sheep and characterized its molecular features. We then evaluated associations between *ID2* polymorphisms and lambing number in Hetian ewes and examined how *ID2* overexpression affects activity and proliferation of primary ovarian GCs.

## 2. Materials and Methods

### 2.1. Test Sample Collection

Hetian sheep ewes were maintained at the Experimental Station of Tarim University under uniform housing and feeding conditions. The feeding regime was as follows: prior to mating and during gestation all ewes were offered high-quality pasture plus a supplementary concentrate/compound feed; during lactation, the feeding allowance was increased with additional succulent forages and protein-rich feed. Drinking water, vaccination and deworming protocols were identical across the flock. Breeding management was by artificial insemination: once estrus was detected (by teaser ram method and external observation of vulvar hyperemia, swelling and mucus discharge), insemination was performed under a uniform protocol. For the cloning of the *ID2* gene, a healthy Hetian ewe in puberty was selected at the Experimental Station and humanely euthanized in accordance with institutional animal care protocols. Immediately after slaughter, the hypothalamus was dissected, snap-frozen in liquid nitrogen and transferred to −80 °C until RNA extraction. For tissue expression profiling, five healthy females of similar body weight were sampled at each of three stages: Prepuberty (approximately 90 days of age), puberty (first detected estrus), and postpuberty (10 days after the first estrus). Selection criteria for these animals were as follows: clinically healthy with no reproductive disorders, body weight within ±10% of the stage mean, no antibiotic or hormone treatments within the previous 30 days. Following humane slaughter, tissue specimens (hypothalamus, pituitary, ovary, oviduct, and uterus) were aseptically excised using sterile scissors and scalpels, minced, transferred to sterile 5 mL cryovials, snap-frozen in liquid nitrogen, and stored at −80 °C until analysis. In sheep reproductive expression profiling, rapid freezing and storage at −80 °C are correct practices to preserve RNA integrity and minimize degradation across developmental or estrous stages [23]. Puberty was defined following the standard for small ruminants. Puberty was identified by twice-daily observations at 10:00 and 16:00 using teaser rams and visual inspection of external signs. Criteria included acceptance of mounting (standing estrus) together with vulvar hyperemia and swelling, and the presence of cervical/vulvar mucus. Behavioral changes were recorded contemporaneously for each ewe.

For population genetic analyses, whole blood was collected from 157 multiparous ewes (3–5 years of age) via jugular venipuncture into EDTA-containing vacuum tubes and stored at −40 °C until DNA extraction. Parity distribution of the sampled ewes (3–5 years of age) was recorded as parity 2, *n* = 62 (39.5%); parity ≥ 3, *n* = 95 (60.5%), total *n* = 157. Of these 157 ewes, 78 delivered twins and 79 delivered singletons; all were bred by artificial insemination and the number of live lambs was recorded within 24 h after parturition.

All animal procedures were approved by the Institutional Animal Care and Use Committee of Tarim University of Science and Technology and complied with the institutional Animal Experiment Guide and applicable national regulations. This study adhered to the ethical guidelines set by the Ethics Committee of Tarim University of Science and Technology (approval number SYXK 2020-009), approved on 23 April 2020.

### 2.2. Ovarian Collection

Healthy Hetian ewes were selected and humanely euthanized; ovarian tissues were immediately collected post-mortem. and the ovaries located on both sides of the uterus were cut off with sterile surgical scissors, rinsed (sprayed) with 75% alcohol for disinfection, and the blood was removed. They were placed in a pre-cooled PBS buffer bottle (pre-cooled with ice packs in a foam incubator) and quickly transferred to a sterile cell culture room within 4–5 h for subsequent operations. The above ovaries were cut off from the excess connective tissue around the ovaries, and then placed in a sterile beaker, added 75% alcohol, and soaked for 30 s. The alcohol was poured out, and the PBS buffer was rinsed 3–5 times.

### 2.3. Primer Design

The *ID2* nucleotide sequence (accession number XM_004017705.5) was retrieved from the NCBI Nucleotide database (https://www.ncbi.nlm.nih.gov) (accessed on 3 September 2025) based on the reference genomic assembly GCF_016772045.2-RS_2023_10A. Subsequently, amplification primers and genotyping primers for the ID2 gene were designed using Primer Premier 5.0 software. Fluorescence quantitative PCR primers were designed according to the CDS of the *ID2* gene of Hetian sheep (Table 1). The *ACTB* gene was used as the reference gene for qPCR analysis, because it is a classical housekeeping gene whose expression has been reported to remain stable in different tissues and under various physiological conditions in sheep [24]. Prior to the main experiment, we confirmed that *ACTB* gene Ct values varied minimally. Target gene expression levels were therefore quantified using the 2^−ΔΔCt^ method with *ACTB* gene as the normalizer.

### 2.4. ID2 Gene Cloning

PCR amplification was carried out with the primers listed in (Table 1). Each 25 µL reaction contained 1.0 µL of ovarian cDNA, 1.0 µL of each forward and reverse primer (10 µM), 12.5 µL 2× EasyTaq^®^ PCR SuperMix (TransGen Biotech Co., Ltd., Beijing, China), and 9.5 µL nuclease-free water. Thermocycling conditions were as follows: initial denaturation at 94 °C for 5 min; 40 cycles of 95 °C for 30 s, 56 °C for 30 s, and 72 °C for 2 min; followed by a final extension at 72 °C for 10 min and a hold at 4 °C. Amplicons were visualized on 1.5% agarose gels to confirm expected size and integrity. purified PCR products were subjected to Sanger sequencing on an Applied Biosystems platform (Sangon Biotech (Shanghai) Co., Ltd., Shanghai, China) to identify sequence variants. Sequence chromatograms were trimmed and aligned using DNAMAN Version 6.0.

### 2.5. Bioinformatics Analysis

The sequencing results were assembled using DNAMAN 6.0, and homology searches were performed using BLAST on the NCBI website (https://blast.ncbi.nlm.nih.gov/Blast.cgi) (accessed on 3 September 2025). The physicochemical properties of the ID2 protein were predicted using the ProtParam website (https://web.expasy.org/protparam) (accessed on 3 September 2025). The hydrophobicity and transmembrane structure of the protein were predicted using Prot Scale software (https://web.expasy.org/protscale/) (accessed on 3 September 2025) and TMHMM 2.0 (https://services.healthtech.dtu.dk/services/TMHMM-2.0/) (accessed on 3 September 2025). Signal P 6.0 (https://services.healthtech.dtu.dk/services/SignalP-6.0/) (accessed on 3 September 2025) and Net Phosphorylation 3.1 (https://services.healthtech.dtu.dk/services/NetPhos-3.1/) (accessed on 3 September 2025) servers were used to predict signal peptides and phosphorylation sites. PSIPRED 4.0 (https://bioinf.cs.ucl.ac.uk/psipred/) (accessed on 3 September 2025) and Phyre 2.0 (http://www.sbg.bio.ic.ac.uk/phyre2/html/page.cgi?id=index) (accessed on 3 September 2025) were used to predict the secondary and tertiary structures of ID2 protein. The amino acid sequence alignment of Hetian sheep ID2 protein with other species was constructed using MEGA 11, and a phylogenetic tree was constructed using the neighbor-joining (NJ) method.

### 2.6. ID2 Gene Tissue Expression

Total RNA was isolated from five ovine reproductive tissues (hypothalamus, pituitary, ovary, oviduct and uterus) at three physiological stages using TRIzol reagent (Invitrogen, Carlsbad, CA, USA). RNA concentration and purity were assessed using a NanoDrop 8000 spectrophotometer (NanoDrop Technologies, Wilmington, DE, USA). cDNA was synthesized using the Takara Reverse Transcription Kit (TaKaRa Bio Inc., Dalian, China) for qPCR assays. Primers were designed as listed in Table 1, and *ACTB* served as the endogenous control for normalization. Expression of *ID2* in reproductive tissues before, during, and after estrus in Hetian sheep was measured on a CFX96 Real-Time PCR Detection System (Bio-Rad Laboratories, Hercules, CA, USA). Each 15 µL RT-qPCR contained 7.5 µL SYBR Green Real-time PCR Mix, 5.5 µL nuclease-free water, 0.5 µL of each primer (10 µM), and 1.0 µL cDNA (500 ng/µL). Cycling conditions were as follows: 94 °C for 30 s; 40 cycles of 94 °C for 20 s, 58 °C for 15 s, and 72 °C for 15 s; followed by melt-curve analysis (95 °C for 15 s, 60 °C for 30 s, 95 °C for 15 s). Melt-curve parameters used the instrument’s default settings. Each sample was run in triplicate.

### 2.7. ID2 Genotype Analysis

A total of 157 ewes of the Hetian sheep breed were sampled for genotyping in this study. All animals were female and belonged to a single commercial flock managed under uniform conditions. No targeted selection of close-kin (such as full-sib or half-sib) individuals was performed; background relatedness may exist as the flock is a nucleus breeding herd. All ewes included had recorded live-born lambs (i.e., sample selection excluded non-lambing individuals). The phenotype used for the SNP–litter size analysis was the number of live-born lambs per lambing (within 24 h of birth) and all ewes in this set had lambing records.

Genomic DNA was extracted from blood by the phenol–chloroform method. Genotyping primers designed for the *ID2* loci were used to detect target variants. PCR was performed on a Veriti™ 96-Well Thermal Cycler (Thermo Fisher Scientific, Waltham, MA, USA). Each 25 µL reaction contained 12.5 µL 2× EasyTaq^®^ PCR SuperMix (TransGen Biotech, Beijing, China), 9.5 µL nuclease-free water, 1.0 µL of each primer (10 µM), and 1.0 µL genomic DNA. Amplicons of the expected size were purified and submitted to Sangon Biotech (Shanghai, China) for Sanger sequencing. Trace files were inspected in Chromas 2.6.6, and sequences were aligned to the ovine reference in DNAMAN to identify polymorphic sites.

### 2.8. Isolation, Culture, and Identification of Ovarian GCs

Fresh, untreated ovaries were rinsed in sterile PBS. Surface follicles were incised with a sterile scalpel, and follicular fluid was allowed to drain into pre-equilibrated collection medium. The cell suspension was centrifuged at 2000 rpm for 5 min. The supernatant was discarded, leaving a white cell pellet. The pellet was resuspended in 2 mL culture medium containing antibiotics (e.g., 3% penicillin–streptomycin), then centrifuged at 1000 rpm for 5 min; this wash was repeated three times to obtain a granulosa-cell pellet. The centrifuged GCs were inoculated into a T25 culture flask containing 7 mL of DMEM medium supplemented with 15% FBS and 1% penicillin-streptomycin. Cells were evenly distributed and incubated at 37 °C in 5% CO_2_ for 24 h, after which cultures were inspected and the medium was refreshed.

GCs purity was verified by FSHR immunofluorescence. Cells were fixed in 4% paraformaldehyde for 15 min, permeabilized with 0.3% Triton X-100 for 10 min, and blocked with 20% BSA for 30 min. Cultures were incubated with anti-FSHR primary antibody for 1 h at room temperature, rinsed, and then incubated with the appropriate fluorophore-conjugated secondary antibody for 30–45 min. Nuclei were counterstained with DAPI for 10 min at 37 °C and imaged on an IX73 inverted fluorescence microscope (Olympus, Tokyo, Japan).

### 2.9. Lentivirus Transfection of Eggs and CCK-8 Assay

A lentiviral overexpression construct for the ovine *ID2* gene (Hanheng Biotech, Shanghai, China) was used to transduce adherent GCs. Cells were seeded in 24-well plates at 1 × 10^5^ cells/well and transduced when cultures reached 2 × 10^5^ cells/well, following the manufacturer’s instructions. After 24 h at 37 °C, the virus-containing medium was replaced with fresh complete medium, and cultures were maintained at 37 °C for an additional 48 or 72 h.

Cell viability was evaluated using the Cell Counting Kit-8 (CCK-8) (Solarbio Science & Technology Co., Ltd., Beijing, China). GCs were seeded in 96-well plates at 2000 cells/well. At 48 and 72 h, 10 µL CCK-8 reagent was added per well and plates were incubated for 2 h at 37 °C. Absorbance was read at 450 nm on a microplate reader (Tecan Group Ltd., Männedorf, Switzerland), and viability was calculated relative to the control group. Assays were performed in triplicate, and results are reported as mean ± SD.

### 2.10. Enzyme-Linked Immunosorbent Assay

Progesterone (P4) and estradiol (E2) in culture supernatants were quantified by ELISA to assess the effect of ID2 overexpression. Supernatants were collected at 48 and 72 h post-transduction, clarified by brief centrifugation, and assayed with commercial ELISA kits (Solarbio Science & Technology, Beijing, China) according to the manufacturers’ instructions. Concentrations were reported as ng/mL (P4) and pg/mL (E2). Each sample was run in triplicate, and the results were compared with the corresponding control group.

### 2.11. RT-PCR Detection of ID2-Related Gene Expression

Total RNA was extracted from *ID2*-overexpressing and control cells using TRIzol reagent (Invitrogen), cDNA was synthesized using the Takara Reverse Transcription Kit (TaKaRa Bio Inc., Dalian, China) for qPCR assays. mRNA levels of predicted ID2 interactors from the STRING database (*ID1*, *ID3*, *NFIL3*, and TCF3) and pathway-related genes (*BMPR2*, *RBX1*, and *SMAD7*) were quantified by RT-qPCR using the primers listed in (Table 2). Expression was normalized to *ACTB* and calculated with the 2^−ΔΔCt^ method. All reactions were run in triplicate.

### 2.12. Data Processing and Statistical Analysis

Relative mRNA expression was calculated using the 2^−ΔΔCt^ method. The population genetic indicators were analyzed using Excel 2019 software, and the linkage disequilibrium results of the mutation sites were statistically analyzed using the SHEsis (http://analysis.bio-x.cn) (accessed on 3 September 2025) online website. One-way ANOVA in SPSS 26.0 software was used to determine the significance, with *p* < 0.01 indicating an extremely significant difference and *p* < 0.05 indicating a significant difference.

(1) Gene frequency and genotype frequency

In a biological (diploid) population, genotype frequencies represent the proportion of individuals carrying each genotype at a locus. If we denote the genotypes as AA, AB, and BB, then one computes:

Genotype frequency: P_AA_ = AA individuals/total number; P_AB_ = AB individuals/total number; P_BB_ = BB individuals/total number

The allele frequencies indicate how common each allele is in the gene pool. Denoting the alleles as A and B, one calculates the following:

Allele frequency: PA = (number of AA individuals + number of AB individuals/2)/total number

PB = (number of BB individuals + number of AB individuals/2)/total number

(2) Hardy–Weinberg equilibrium test: $x^{2}=\sum_{i-1}^{m}\frac{{\left(f_{i}-{\mathrm{np}}_{i}\right)}^{2}}{{\mathrm{np}}_{i}}$.
(3) Population homozygosity (*H*_o_) test: $Ho=\sum_{i-1}^{n}p_{i}^{2}$.
(4) Population heterozygosity (*H*_e_) test: $He=1-\sum_{i=1}^{n}p_{i}^{2}$.
(5) Effective allele (*N*_e_) test: $\mathrm{Ne}=\frac{1}{\sum_{i=1}^{n}p_{i}^{2}}$.
(6) Polymorphic Information Content (PIC) Test: $\mathrm{PIC}=1-\sum_{i=1}^{n}p_{i}^{2}-\sum_{i=1}^{n-1}\sum_{j=i+1}^{n}2p_{i}^{2}p_{j}^{2}$.

## 3. Test Results and Analysis

### 3.1. Cloning and Nucleotide Sequence Analysis of the ID2 Gene of Hetian Sheep

**Figure 1:**
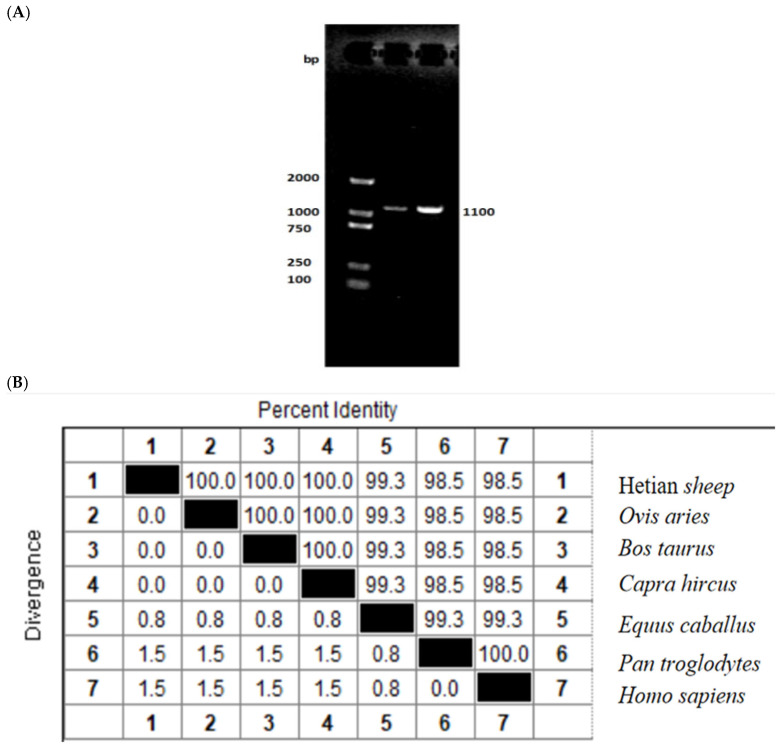

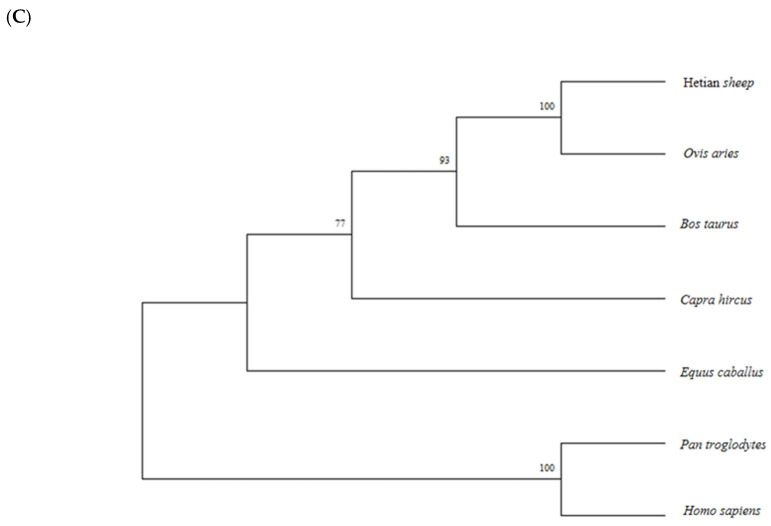
Cloning and nucleotide sequence analysis of Hetian sheep ID2 gene. (**A**) Detection of the coding sequence of the Hetian sheep *ID2* clone via agarose gel electrophoresis. (**B**) Nucleotide homology comparison between the sequences obtained in this study and ID2 sequences from other animal species. (**C**) Neighbor-joining tree of the sequences obtained in this study and the ID2 sequences identified in other animal species. The cloned sequences in this study are shown in a box, and the ID2 sequences from other animal species include sheep (accession number: XP_004005720.1), goat (accession number: XP_005687088.1), cattle (accession number: NP_001029403.1), horse (accession number: XP_001503661.1), orangutan (accession number: XP_034808448.1), and human (accession number: NP_002157.2).

Total RNA was extracted from the hypothalamic tissue of Hetian sheep, reverse-transcribed into cDNA, and the coding region of the *ID2* gene was subsequently amplified from this cDNA.A specific amplicon of 1100 bp was obtained, and the coding sequence (CDS) spanned 405 bp (Figure 1A). The sequence matched the predicted ovine *ID2* mRNA (GenBank accession XM_004005671.4). At the amino-acid level, the Hetian sheep ID2 protein was identical to those of sheep, goat, and cattle (100% identity) and highly conserved relative to horse (99.3%), gorilla (98.5%), and human (98.5%) (Figure 1B). Phylogenetic analysis grouped Hetian sheep ID2 closest to other ruminants (sheep, cattle, goat), followed by horse and gorilla, with human most distant among the species compared (Figure 1C). These findings confirm that the sequence obtained corresponds to the ovine *ID2* gene.

### 3.2. Prediction and Analysis of the Characteristics of Hetian Sheep ID2 Protein

**Figure 2:**
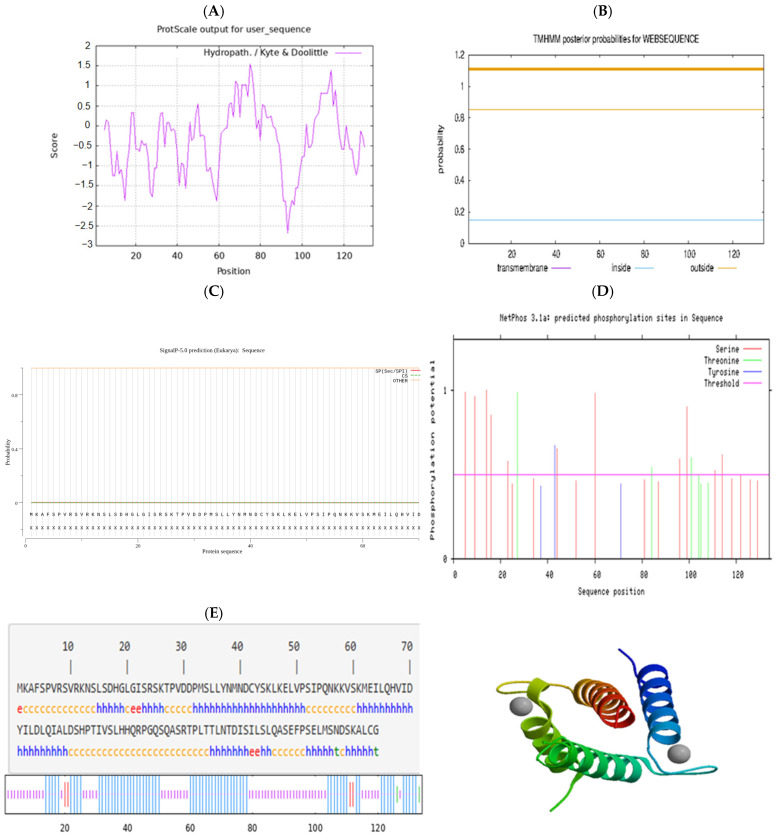
Characteristic analysis of sheep ID2 protein. (**A**) Hydrophilicity analysis of Hetian sheep ID2 protein. (**B**) Prediction of the transmembrane structure of Hetian sheep ID2 protein. (**C**) Prediction of the signal peptide of Hetian sheep ID2 protein. (**D**) Analysis of phosphorylation sites of Hetian sheep ID2 protein. (**E**) Prediction of secondary and tertiary structures of Hetian sheep ID2 protein.

The physicochemical properties of the protein of the Hetian sheep *ID2* gene were interpreted, indicating that the molecular mass of the protein was 14,860.06 u, the theoretical isoelectric point pi was 7.82, and the molecular formula was C_648_H_1061_N_181_O_203_S_7_. The total number of atoms was 2100, encoding a total of 134 amino acids, of which there were 20 serine (S) and 17 leucine (L), accounting for 14.9% and 12.7%, respectively, and no tryptophan (W) was found. The total number of negatively charged amino acid residues (Asp and Glu) was 13. The total number of positively charged amino acid (Arg + Lys) residues was 14, and the instability coefficient was 61.72 (Table 3). The hydrophobicity prediction of the ID2 protein is shown in (Figure 2A), indicating that the ID2 protein is hydrophilic. The transmembrane domain of Hetian sheep ID2 protein was predicted, and the results showed that ID2 protein did not contain a transmembrane domain (Figure 2B). By predicting the signal peptide information of the Hetian sheep ID2 protein, it was found that the ID2 protein did not have a signal peptide and was not a secretory protein (Figure 2C). Hetian sheep ID2 protein has a total of 29 phosphorylation sites, including 20 serine (S) sites, 6 threonines (T) sites, and 3 tyrosine (Y) sites (Figure 2D). The secondary structure prediction of ID2 protein showed that the protein contained 67 α-helices (50%), 5 extended chains (3.73%), 2 β-folds (1.49%), and 60 irregular coils (46.78%); α-helices and irregular coils were spread throughout the amino acid chain, with only extended chains and a few β-folds (Figure 2E).

### 3.3. Analysis of the Expression of the ID2 Gene in Different Tissues of Hetian Sheep at Different Puberty Stages

**Figure 3:**
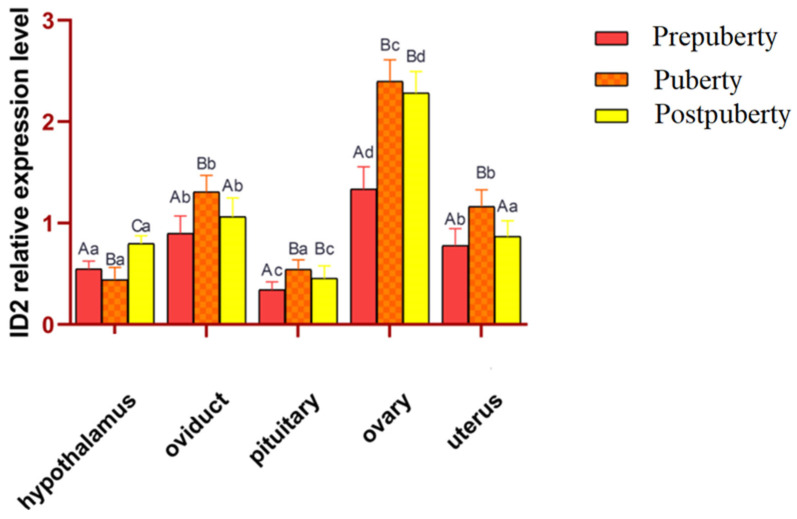
Relative expression levels of *ID2* gene in different tissues of Hetian sheep at different stages of puberty. (1) Values of the same tissues and different periods with different capital letter superscripts indicate significant differences (*p* < 0.05), whereas with the same capital letter or no letter superscripts, there was no significant difference (*p* > 0.05). (2) Values of the same period, different tissues with different small letter superscripts indicate a significant difference (*p* < 0.05), while with the same small letter superscripts, there was no significant difference (*p* > 0.05).

Real-time fluorescence quantitative PCR for the expression of the *ID2* gene in five tissues of Hetian sheep, including the hypothalamus, oviduct, pituitary, ovary, and uterus, are shown in (Figure 3). The *ID2* gene was expressed in five tissues in the three periods before puberty, during puberty, and after puberty. The expression level of the *ID2* gene in the ovary was significantly higher than that in other tissues in the three periods (*p* < 0.05). The expression levels of the oviduct, pituitary, and uterus in puberty were significantly higher than those in other periods (*p* < 0.05). The expression level of the hypothalamus decreased significantly from Prepuberty to puberty (*p* < 0.05) and increased significantly from puberty to Postpuberty (*p* < 0.05).

Notably, *ID2* gene expression was highest in the ovary during puberty, significantly higher than in other tissues (e.g., hypothalamus, pituitary) at the same stage.

### 3.4. Descriptive Characteristics of the Genotyped Ewe Population

To provide context for the subsequent SNP–litter size association analyses, Table 4 summarizes the key descriptive statistics of the 157 genotyped ewes. It includes information on sample size, age, parity distribution, flock origin, mating method, live-born lamb counts and reproductive parameters (fertility, fecundity, prolificacy).

The results showed that all animals were female, from a single commercial flock under uniform management, with a mean age of 3.4 ± 0.7 years (range 3.0–5.0). All 157 ewes lambed, yielding a total of 236 live-born lambs, an average of 1.50 ± 0.50 lambs per ewe. Additional reproductive metrics include a fertility of 100.0% (157/157), fecundity of 150.3%, and a prolificacy rate (≥2 lambs) of 50.3%.

### 3.5. ID2 Gene PCR Amplification Results and Polymorphic Site Peak Diagram

**Figure 4:**
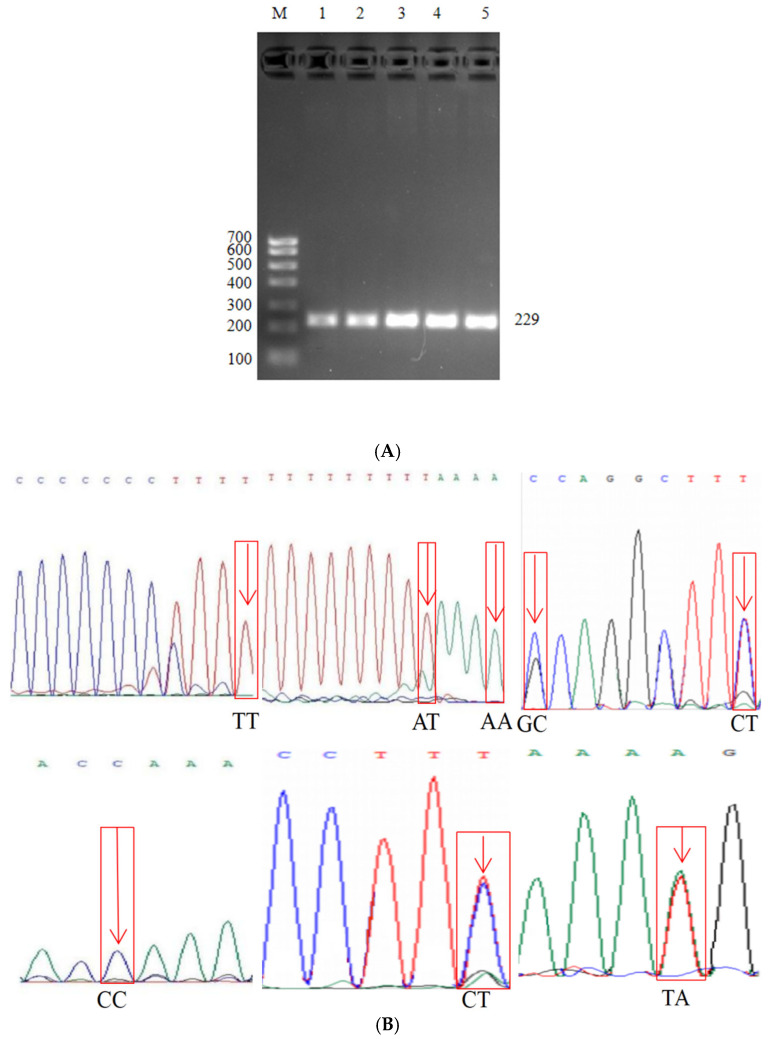
*ID2* gene polymorphic sites. (**A**) *ID2* gene PCR amplification target fragment. M: DNA marker; Lanes 1–5: PCR products, showing a specific band at 207 bp, indicating successful amplification of the target fragment (**B**) *ID2* gene SNP site genotype sequencing diagram. confirming the existence of genetic polymorphism in the *ID2* gene. An arrow highlights the key mutation site.

Through PCR amplification, a specific band of 207 bp was obtained (Figure 4A). The amplified product was sent to (Sangon Biotech) for sequencing, and a total of 8 mutation sites were found (Figure 4B).

### 3.6. Genetic Analysis of SNP Variant Sites in ID2 Gene

A total of eight polymorphic sites were detected in the *ID2* gene in Hetian sheep, among which three sites (g.18202372 G>A, g.18202426 C>T, and g.18202472 G>C) had three genotypes, while the other five sites had two genotypes. The frequencies of the polymorphic sites are shown in Table 5. Hardy–Weinberg analysis revealed that the genotype distribution of all variant sites conformed to Hardy–Weinberg equilibrium (*p* > 0.05).

### 3.7. SNP and Linkage Disequilibrium of ID2 Gene

**Figure 5:**
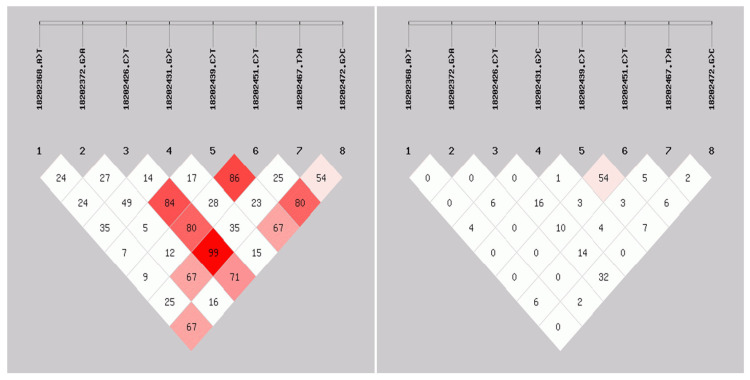
Linkage disequilibrium analysis of SNP mutation sites in *ID2* gene.

According to (Table 6), the g.18202368 A>T, g.18202372 G>A, g.18202426 C>T, g.18202451 C>T, and g.18202472 G>C sites of the *ID2* gene are moderately polymorphic (0.25 < PIC < 0.5), and the g.18202431 G>C and g.18202467 T>A sites are lowly polymorphic (PIC < 0.25). The results of linkage disequilibrium analysis of SNP mutations in the ID2 gene are shown in Figure 5. The r^2^ value of the linkage between g.18202439 C>T and g.18202451 C>T is 0.54, indicating that the two mutation sites are strongly linked to each other (D′ > 0.86, r^2^ > 0.30), and there is no obvious linkage between the other sites.

### 3.8. Association Analysis Between ID2 Gene Polymorphism and Litter Size

*ID2* genotypes were tested for association with litter size; values are presented as mean ± SD and results are summarized in (Table 7). At g.18202368 A>T, AA individuals had a significantly higher mean litter size than AT individuals (*p* < 0.05). At g.18202372 G>A, AA individuals showed a highly significant increase in mean litter size compared with GG individuals (*p* < 0.01). At g.18202431 G>C, GC individuals had a higher mean litter size than GG individuals (*p* < 0.05). At g.18202472 G>C, CC individuals had a higher mean litter size than GG individuals (*p* < 0.05).

### 3.9. Identification of Ovarian GCs

**Figure 6:**
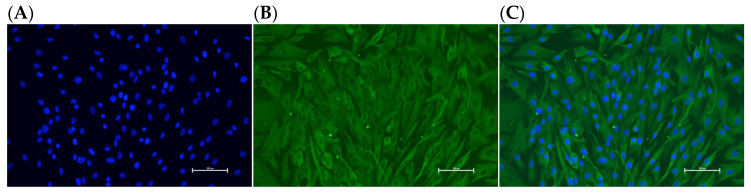
Identification of ovarian GCs in Hetian sheep. (**A**) DAPI-stained nuclei (blue); (**B**) FSHR-specific signal (green); (**C**) merged image. Positive cytoplasmic FSHR confirms GCs’ identity. Magnification: 200×.

The purity of the cells exceeds 90%, indicating that they are ovarian GCs. To confirm that the isolated cells were GCs, immunofluorescent staining was performed to detect FSHR expression. It was found that the GCs isolated from the ovaries of Hetian sheep specifically expressed FSHR, as shown in (Figure 6). The proportion of positive cells exceeded 95% (Figure 6C), indicating that the isolated cells were GCs.

### 3.10. ID2 Overexpression Enhances Proliferation of Ovarian GCs

**Figure 7:**
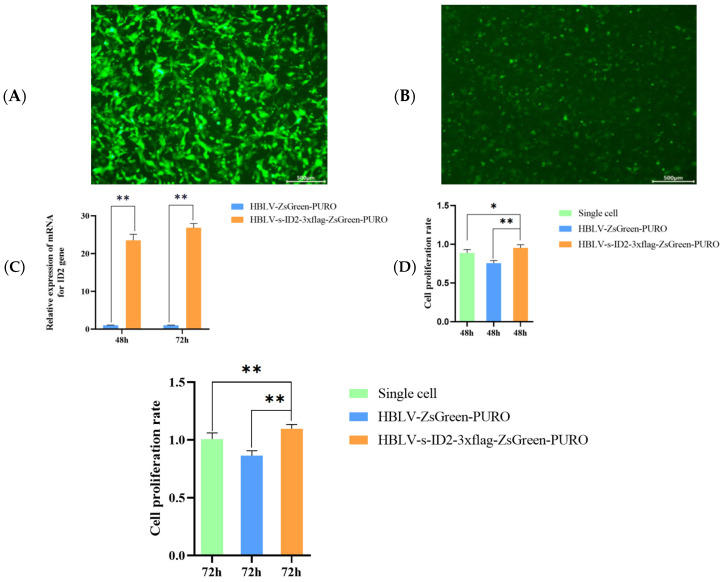
*ID2* is overexpressed in sheep ovarian GCs (scale bar: 500 µm). (**A**) Fluorescence image of GCs transfected with the *ID2* overexpression construct. (**B**) Fluorescence image of the negative control from empty vector transfection. (**C**) mRNA overexpression of *ID2* gene in ovarian GCs. (**D**) Effect of overexpression of *ID2* gene on proliferation of ovarian GCs. indicates extremely significant difference (*p* < 0.01), indicates significant difference (*p* < 0.05). * indicates *p* < 0.05, ** indicates *p* < 0.01.

The *ID2* gene was measured at 48 h and 72 h. Ovarian GCs showed a large amount of green fluorescent protein under the excitation of the blue light source of the fluorescence microscope Figure 7A,B. The expression of the *ID2* gene increased with the increase in transfection time. After *ID2* overexpression, the results of qRT-PCR showed that the mRNA level of the ID2 gene transfection group at 48 h and 72 h transfection was significantly higher than that of the blank group (*p* < 0.01), and the expression level gradually increased with time Figure 7C. The CCK-8 results are shown in Figure 7D. As time increases, it can be seen that the overexpression of the *ID2* gene has a certain promoting effect on the proliferation of ovarian GCs (*p* < 0.01), which preliminarily proves that the *ID2* gene can promote the proliferation of ovarian GCs.

### 3.11. Effects of ID2 Overexpression on P4 and E2 Hormone Production

**Figure 8:**
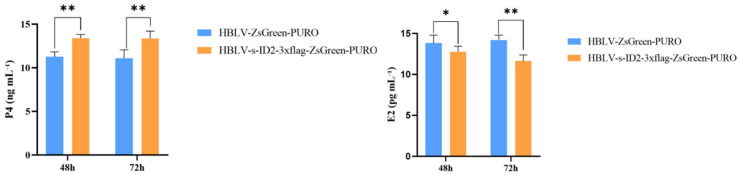
Effect of *ID2* overexpression on E2 and P4 concentrations. * indicates *p* < 0.05, ** indicates *p* < 0.01.

To evaluate the effect of *ID2* on reproductive hormones, the expression levels of P4 and E2 were studied. The results are shown in (Figure 8). The effect of *ID2* overexpression on P4 concentration was significantly higher than that of the empty load group at 48 h and 72 h (*p* < 0.01); The effect of ID2 overexpression on E2 concentration was significantly lower than that of the empty load group at 48 h (*p* < 0.05), and significantly lower than that of the empty load group at 72 h (*p* < 0.01).

### 3.12. Effects of ID2 Gene Overexpression on Reproduction-Related Genes

**Figure 9:**
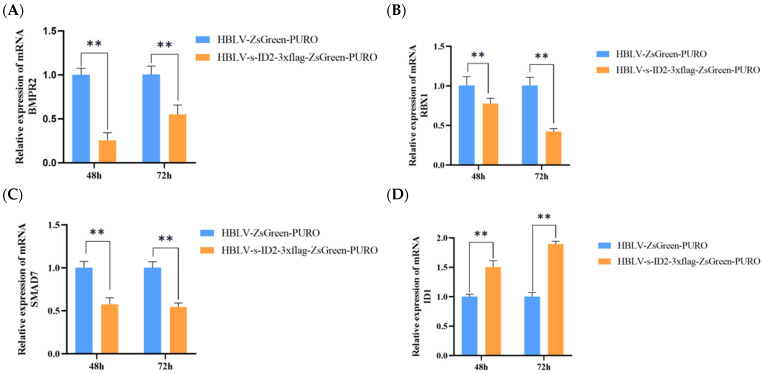

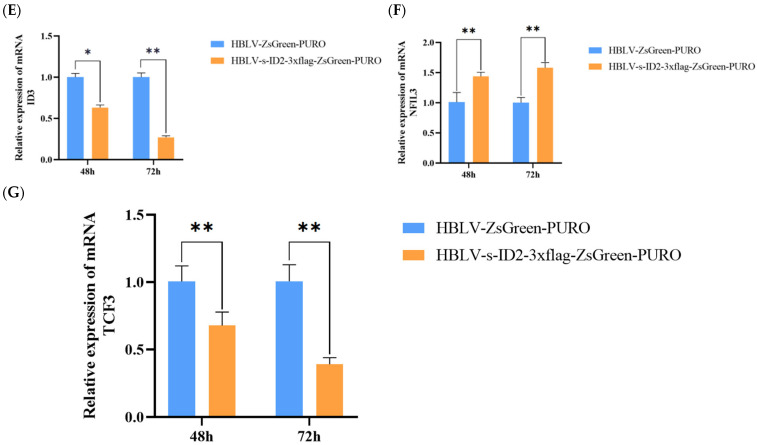
Effects of *ID2* overexpression on the relative mRNA expression of *BMPR2*, *RBX1*, *SMAD7*, *ID1*, *ID3*, *NFKL3*, and *TCF3* in GCs at 48 h and 72 h post-transfection. (**A**) represents the mRNA expression level of *BMPR2* at 48 h and 72 h after *ID2* overexpression. (**B**) represents the mRNA expression level of *RBX1* at 48 h and 72 h after *ID*2 overexpression. (**C**) represents the mRNA expression level of *SMAD7* at 48 h and 72 h after *ID2* overexpression. (**D**) represents the mRNA expression level of *ID1* at 48 h and 72 h after *ID2* overexpression. (**E**) represents the mRNA expression level of *ID3* at 48 h and 72 h after *ID2* overexpression. (**F**) represents the mRNA expression level of *NFKL3* at 48 h and 72 h after *ID2* overexpression. (**G**) represents the mRNA expression level of *TCF3* at 48 h and 72 h after *ID2* overexpression. Bars represent mean ± SEM (*n* = 3 biological replicates). * indicates *p* < 0.05, ** indicates *p* < 0.01 (Independent Samples *t*-test).

*ID2* gene overexpression was collected for 48 h and 72 h, and RNA was extracted and reverse transcribed to synthesize c DNA. The relative expression levels were measured by qRT-PCR using the same signaling pathway genes of *ID2* (*BMPR2*, *RBX1*, *SMAD7*) and the *ID2*-related genes (*ID1*, *ID3*, *NFIL3*, *TCF3*) found by STRING. The results are shown in (Figure 9). The relative expression levels of *BMPR2*, *RBX1*, and *SMAD7* genes in the empty group were significantly higher than those in the overexpression group (*p* < 0.01), the *BMPR2* overexpression group at 48 h was significantly lower than that at 72 h (*p* < 0.01) (Figure 9A), the *RBX1* overexpression group was significantly higher than that at 48 h (*p* < 0.01) (Figure 9B), and the *SMAD7* overexpression group had no significant changes at 48 and 72 h (Figure 9C). The relative expression level *of ID1* in the overexpression group was significantly higher than that in the empty load group (*p* < 0.01) (Figure 9D), the relative expression level of *ID3* in the overexpression group was significantly lower than that in the empty load group at 48 h (*p* < 0.01) (Figure 9E), and the relative expression level of *NFIL3* in the overexpression group was significantly higher than that in the empty load group (*p* < 0.01) (Figure 9F), and the relative expression level of *TCF3* in the overexpression group was significantly lower than that in the empty load group (Figure 9G).

## 4. Discussion

In this study, we cloned and characterized the coding sequence (CDS) of the *ID2* gene in Hetian sheep and examined its role in ovarian GCs function and reproductive performance. The high homology of ovine *ID2* with its orthologs in sheep, goats, and cattle underscores strong evolutionary conservation and supports a functionally relevant role in mammalian reproductive physiology. These findings provide new insight into molecular mechanisms underlying follicular development and fertility in sheep.

Amino-acid composition analysis showed that the Hetian sheep ID2 protein contains relatively high proportions of serine and leucine. Serine is polar, whereas leucine is hydrophobic; nonetheless, the overall bioinformatics profile predicts a hydrophilic protein, consistent with the distribution of residues and domain architecture. Prior work indicates that serine availability can promote oocyte growth and maturation, with cumulus cells supplying serine to enclosed oocytes via gap junctions [25,26]. In parallel, leucine-rich repeat signaling components such as LGR5 are expressed in the ovary and have been implicated in successful pregnancy [27]. While these observations do not imply a direct causal link between ID2 residue frequencies and ovarian signaling, they are congruent with a broader role for amino-acid–sensitive and leucine-rich pathways in reproductive biology.

RT-qPCR demonstrated tissue-specific expression of *ID2* in Hetian sheep, with the highest levels in the ovary and the lowest in the pituitary. This differential pattern is consistent with a role in ovarian development and maturation, a conclusion further supported by the effects of *ID2* on GCs viability and proliferation. *ID2* expression was also relatively high in the uterus, suggesting additional functions in female reproductive processes.

We investigated associations between *ID2* polymorphisms and litter size in 157 Hetian ewes using PCR and direct sequencing. Several single-nucleotide polymorphisms (SNPs) showed significant associations with litter size, notably g.18202368 A>T, g.18202372 G>A, g.18202431 G>C, and g.18202472 G>C. These results suggest that specific *ID2* variants are linked to fecundity in Hetian sheep. Given the moderate sample size (*n* = 157 ewes) in the present genotyping analysis, the possibility of sampling error cannot be excluded. Genetic association studies indicate that smaller sample sizes reduce statistical power and may increase the risk of both false negatives and less reliable effect-size estimates. Accordingly, validation of the identified polymorphisms in larger, and independent sheep populations will be necessary to determine whether these SNPs can serve as reliable adjunct markers for selection and to clarify the relationship between allele frequencies and reproductive performance.

The ovary not only supports folliculogenesis and ovulation but also regulates the estrous cycle and follicular homeostasis through steroid hormones such as E2 and P4, thereby shaping reproductive outcomes. During follicle growth, GCs are essential for maintaining the intrafollicular microenvironment by coordinating the secretion of hormones and paracrine factors. Transforming growth factor-β (TGF-β) is a pleiotropic cytokine that controls diverse cellular processes [28]. TGF-β family ligands are intimately involved in ovulation, fertilization, and the establishment and maintenance of pregnancy; even early reproductive events—including male and female germline specification—are regulated by TGF-β–related proteins [29]. Inhibitory SMADs (SMAD6/7) act as intracellular antagonists. In particular, SMAD7 serves as a critical negative regulator of TGF-β/BMP signaling by competing with receptor-regulated SMADs, thereby influencing oocyte–somatic-cell communication and GCs function [30]. Manipulating SMAD7 expression alters follicular development in mice [31], and SMAD7 has been implicated in TGF-β–induced apoptosis, affecting folliculogenesis. Moreover, SMAD7-mediated signaling can attenuate TGF-β dependence on *ID2* [32]. Because the Id gene family comprises major downstream targets of BMP/SMAD signaling, decreases in *ID2* expression may suppress TGF-β activity. Consistent with this, Juliano et al. [33] identified *ID2* as a predicted target of exosomal miRNAs regulating TGF-β/BMP components in mares; *ID2* was detected in GCs and in follicular-fluid exosomes at mid-estrus and preovulatory stages. In addition, BMPR2, a type II TGF-β receptor with serine/threonine kinase activity, has been reported to regulate germ-cell activity and to activate *ID2* [34]. BMPR2 supports ovarian development and GCs proliferation [35] and plays essential roles in follicular growth and function across mammals and birds [36]. Beyond the TGF-β superfamily, *ID2* may crosstalk with PI3K/AKT pathways that transduce gonadotropin and growth-factor signals to promote GCs proliferation, inhibit apoptosis, and support oocyte maturation [37,38]. Although prior studies have connected *ID2* to TGF-β superfamily signaling in follicular regulation, its specific functions in Hetian-sheep GCs have been scarcely explored, motivating the present work.

We constructed a lentiviral *ID2* overexpression vector and transduced primary ovine GCs. *ID2* mRNA and protein levels were markedly elevated at 48 h and 72 h relative to empty-vector controls, accompanied by increased cell proliferation, consistent with *ID2*’s mitogenic potential in follicular growth and maturation. ELISA assays showed higher P4 and lower E2 following *ID2* overexpression, indicating involvement in luteinization and corpus luteum function [39]. These hormone shifts align with evidence that steroidogenic profiles during folliculogenesis are tightly controlled by transcriptional and signaling networks, including the TGF-β superfamily [40]. CCK-8 assays further confirmed significantly greater proliferation in the *ID2* group at both time points versus controls.

To probe pathway effects, we quantified transcripts of *ID1*, *ID3*, *NFIL3*, *TCF3*, *BMPR2*, *RBX1*, and *SMAD7* in GCs. *ID2* overexpression significantly upregulated *ID1* and *NFIL3* and downregulated *ID3* and *TCF3*. Within the TGF-β axis, *BMPR2*, *RBX1*, and *SMAD7* were all reduced. Kowanetz et al. [41] reported that chronic TGF-β exposure suppresses *ID2*/*ID3*, whereas BMP-7 induces them in U-2 OS cells, illustrating a bidirectional responsiveness of *ID2/ID3* within the TGF-β/BMP network. Our results are consistent with *ID2* functioning not only downstream of BMP signaling but also feeding back on the TGF-β/BMP pathway. As an I-Smad, SMAD7 antagonizes TGF-β/BMP signaling by competing for receptor binding and recruiting SMURF1/2 to promote R-Smad degradation, establishing a negative feedback loop [42]. In a chondrogenesis model, ID2 overexpression enhanced BMP signaling by suppressing SMAD7, regulating postnatal cartilage formation—mechanistically echoing our observation of SMAD7 downregulation with ID2 overexpression [43]. Notably, RBX1, a core subunit of SCF-type E3 ubiquitin ligases that can target Smad proteins for degradation, was also decreased, further suggesting that ID2 influences the proteostatic control of Smad signaling components. Finally, the observed upregulation of ID1 and NFIL3 agrees with reports that NFIL3 can directly regulate ID2 expression [44], pointing to an interconnected transcriptional circuit.

In summary, *ID2* overexpression modulates GCs fate through TGF-β superfamily–associated pathways: on one hand, suppression of *BMPR2* and *SMAD7* is consistent with enhanced proliferation and luteinization; on the other, coordinated changes in downstream transcription factors—*ID1*/*ID3*, *TCF3*, and *NFIL3*—reshape gene-expression programs that support follicular growth and corpus luteum function.

## 5. Conclusions

In this study, we successfully cloned the *ID2* gene in Hetian sheep, characterized its protein features, and examined its expression across gonadal tissues. The results showed that *ID2* was highly expressed in ovarian tissues, especially during the pubertal stage. We identified four SNP loci in *ID2* (g.18202368, g.18202372, g.18202431, g.18202472) that are significantly associated with lambing number. Furthermore, in vitro overexpression experiments showed that *ID2* promotes the proliferation of sheep granulosa cells, enhances steroid hormone secretion, and likely acts via modulation of the TGF-β and BMP/SMAD signaling pathways. In conclusion, this study provides a new theoretical basis for understanding the proliferation of sheep GCs and the regulation of lambing performance.

## Figures and Tables

**Table 1:** Gene cloning and quantitative PCR primers.

Genes	Primer Sequence (5′→3′)	Amplicon Size (bp)	Annealing Temperature (°C)
ID2	F: CTTCCCTCCTCCCAATCGCT R: GCAGGCAATCACCATTCAATAAAC	1100	56
ID2-1	F: GCTGTGGAAGGAGTCGTGT R: GCGCTTTGCTGTCATTTG	207	58
QID2	F: TCCAGTGAGGTCCGTTAGG R: TGATGTCGGTGTTGAGGGT	317	58
ACTB	F: GCAGATGTGGATCAGCAAGC R: TCTCGTTTTCTGCGCAAGTT	113	58

**Table 2:** Primer design for ID2-related genes.

Genes	Primer Sequence (5′→3′)	Amplicon Size (bp)	Annealing Temperature (°C)
ID1	F: CCAGAACCGCAAGGTGAGCA R: CGCCGATTTCGCCATTGA	161	58
ID3	F: CTCTGGCTTTCTCCTTCCTT R: CGAGTAGCAGTGGTTCATGTC	195	58
RBX1	F: CAGCGATGGATGTGGATACC R: CTCGCTGGCTCAAAACACG	370	58
BMPR2	F: GCAGGTTCTGGTGTCTAGGG R: GTGACAGGTTGCGCTCATTC	251	58
SMAD7	F: GTGCTCAAGAAACTGAAGGAAC R: TCGCAGAGTCGGCTAAGGT	314	58
NFIL3	F: CCGAAGACTTGACGACAGG R: GCCGCTTTTCCCAATACAT	135	58
TCF3	F: AAGCCACGGGACTATTTTG R: GTGCTGAACAACAGGAACTTTA	180	58
ACTB	F: GCAGATGTGGATCAGCAAGC R: TCTCGTTTTCTGCGCAAGTT	133	58

**Table 3:** Amino acid composition of Hetian sheep ID2 predicted protein.

Amino Acids	Quantity	Proportion (%)	Amino Acids	Quantity	Proportion (%)
Alanine (A)	5	3.7%	Leucine (L)	17	12.7%
Arginine (R)	5	3.7%	Lysine (K)	9	6.7%
Asparagine (N)	6	4.5%	Methionine (M)	5	3.7%
Asparticacid (D)	9	6.7%	Phenylalanine (F)	2	1.5%
Cysteine (C)	2	1.5%	Proline (P)	9	6.7%
Glutamine (Q)	7	5.2%	Serine (S)	20	14.9%
Glutamate (E)	4	3.0%	Threonine (T)	6	4.5%
Glycine (G)	4	3.0%	Tryptophan (W)	0	0%
Histidine (H)	5	3.7%	Tyrosine (Y)	3	2.2%
Isoleucine (I)	9	6.7%	Valine (V)	7	5.2%

**Table 4:** Descriptive statistics and reproductive parameters of the genotyped Hetian ewe population.

Variable	Value	Notes
Sample size	157	All genotyped animals included in the SNP–litter size analysis; all selected ewes delivered live lamb(s).
Sex	Female (100%)	All ewes with lambing records.
Herd origin	Single commercial flock	Same farm and uniform management to minimize environmental heterogeneity.
Age at sampling	Mean ± SD: 3.4 ± 0.7; Range: 3.0–5.0	Age recorded at sampling.
Parity distribution	1st: 45 (28.7%); 2nd: 62 (39.5%); ≥3: 50 (31.8%)	Parity = number of previous lambings at sampling.
Mating method	artificial insemination	artificial insemination
Ewes lambed	157 (100.0%)	All sampled ewes delivered live lamb(s).
Total live-born lambs	236 (total)	Sum of live lambs born within 24 h across 157 ewes.
Lambs per ewe	1.50 ± 0.50	Calculated as 236/157 ≈ 1.50. (236/157 ≈ 1.50)
Litter size distribution	Single (1 lamb): 78 (49.7%) Twin (2 lambs): 79 (50.3%) Triplet (3 lambs): 0 (0%)—none observed	Categories based on live-born lambs within 24 h; totals sum to 157 ewes and 236 lambs.
Fertility	100.0%	Fertility = proportion of mated ewes that delivered live lamb(s).
Fecundity	150.3%	Fecundity = total live lambs ÷ number of mated ewes × 100 = (236/157) × 100 ≈ 150.3%.
Prolificacy	50.3%	Prolificacy reported both as mean lambs per lambed ewe (1.50) and proportion of ewes producing multiples (50.3%).

**Table 5:** Gene frequency and genotype frequency at different loci of the *ID2* gene in Hetian sheep.

Location	Genotype	Genotype Frequency (Sample Size)	Allele	Allele Frequency	X^2^ (*p*-Value)
g.18202368 A>T	AA	0.96	A	0.98	0.08 (*p* > 0.5)
	AT	0.04	T	0.02	
g.18202372 G>A	GG	0.59	G	0.77	0.85 (*p* > 0.5)
	GA	0.37	A	0.23	
	AA	0.04			
g.18202426 C>T	CC	0.71	C	0.84	0.37 (*p* > 0.5)
	CT	0.26	T	0.16	
	TT	0.03			
g.18202431 G>C	GG	0.87	G	0.94	0.73 (*p* > 0.5)
	GC	0.13	C	0.06	
g.18202439 C>T	CC	0.93	C	0.96	0.21 (*p* > 0.5)
	CT	0.07	T	0.04	
g.18202451 C>T	CC	0.95	C	0.97	0.11 (*p* > 0.5)
	CT	0.05	T	0.03	
g.18202467 T>A	TT	0.96	T	0.98	0.08 (*p* > 0.5)
	TA	0.04	A	0.02	
g.18202472 G>C	GG	0.58	G	0.77	2.85 (*p* > 0.5)
	GC	0.38	C	0.23	
	CC	0.04			

**Table 6:** Polymorphic information content, heterozygosity, and number of effective alleles at different loci of the *ID2* gene in Hetian sheep.

Location	Genetic Homozygosity	Genetic Heterozygosity	Polymorphic Information Content	Effective Number of Alleles
g.18202368 A>T	0.96	0.43	0.42	1.04
g.18202372 G>A	0.65	0.35	0.29	1.54
g.18202426 C>T	0.73	0.27	0.23	1.37
g.18202431 G>C	0.88	0.12	0.11	0.13
g.18202439 C>T	0.93	0.68	0.65	1.07
g.18202451 C>T	0.95	0.50	0.50	1.05
g.18202467 T>A	0.96	0.04	0.04	1.04
g.18202472 G>C	0.65	0.35	0.29	1.53

**Table 7:** Association analysis between *ID2* gene SNP loci and lamb size in Hetian sheep.

Location	Genotype	Sample Size	Number of Lambs Litter Size
g.18202368 A>T	AA	150	1.52 ± 0.501 ^a^
	AT	7	1.14 ± 0.378 ^b^
g.18202372 G>A	GG	92	1.27 ± 0.447 ^A^
	GA	59	1.83 ± 0.378 ^B^
	AA	6	1.83 ± 0.408 ^B^
g.18202426 C>T	CC	112	1.47 ± 0.501
	CT	40	1.56 ± 0.502
	TT	5	1.8 ± 0.447
g.18202431 G>C	GG	137	1.47 ± 0.501 ^a^
	GC	20	1.75 ± 0.444 ^b^
g.18202439 C>T	CC	146	1.49 ± 0.502
	CT	11	1.73 ± 0.467
g.18202451 C>T	CC	149	1.49 ± 0.502
	CT	8	1.75 ± 0.463
g.18202467 T>A	TT	150	1.51 ± 0.501
	TA	7	1.29 ± 0.488
g.18202472 G>C	GG	92	1.41 ± 0.495 ^a^
	GC	59	1.61 ± 0.492 ^b^
	CC	6	1.83 ± 0.408 ^b^

Note: If the sample size is less than three, no analysis will be conducted; In the same column of data, different uppercase letters on the shoulder indicate significant differences (*p* < 0.01), different lowercase letters on the shoulder indicate significant differences (*p* < 0.05), and the same or no letters on the shoulder indicate no significant differences (*p* > 0.05).

## Data Availability

The raw data supporting the conclusions of this article will be made available by the authors on request.
